# It's not Ebola … it's the systems

**DOI:** 10.9745/GHSP-D-14-00186

**Published:** 2014-12-02

**Authors:** Victor K Barbiero

**Affiliations:** a Global Health: Science and Practice, Associate Editor.

## Abstract

The 2014 Ebola outbreak in West Africa demonstrates key deficiencies in investment in health systems. Despite some modest investment in health systems, our field has instead largely chosen to pursue shorter-term, vertical efforts to more rapidly address key global health issues such as smallpox, polio, malaria, and HIV/AIDS. While those efforts have yielded substantial benefits, we have paid a price for the lack of investments in general systems strengthening. The Ebola deaths we have seen represent a small portion of deaths from many other causes resulting from weak systems. Major systems strengthening including crucial nonclinical elements will not happen overnight but should proceed in a prioritized, systematic way.

## BACKGROUND

The sensational media coverage of Ebola virus disease (EVD) has turned world attention to the crisis in West Africa and the potential spread of EVD to other African countries and beyond. Ebola has been responsible for not only substantial numbers of deaths but also major disruptions to the entire health systems and economies of the countries involved.

While EVD is in the limelight, we should also consider the epidemiology and disease burdens that drive most of the mortality and morbidity in low- and middle-income countries. For example, each month about 66,000 children die from diarrhea worldwide.^1^ Thus, an investment in broader systems strengthening makes sense. Poor health systems, inadequate access to skilled and equipped providers, and the challenges of motivating positive behavior change compound the difficulty of control efforts for Ebola, diarrhea, and other diseases. But systems are neither perfect nor foolproof. The recent Ebola cases in Dallas, Texas, are a testimony that weaknesses exist even in the most sophisticated systems. However, strong systems will mitigate epidemic outbreaks and reduce endemic disease burdens. Strong systems save lives.

Global health leaders have called for major efforts to stop the 2014 EVD outbreak. All recommendations focus on infection containment and therapy.^2^ All have an underlying investment in systems strengthening and behavior change. Jim Yong Kim, President of the World Bank, stated, *“Now we are witnessing the results of our acceptance of the status quo,”* and has urged a commitment to systems strengthening.^3^ He outlines a 4-point strategy consisting of:

1. Support to health workers
2. Improved clinical and diagnostic infrastructure
3. Expanded information, education, and communication (IEC)
4. General infrastructure support

If the Bank's commitment is over the long term, it represents a sea change in global health programming and will promote stronger routine prevention and care across low- and middle-income countries. The Centers for Disease Control and Prevention likewise endorses a longer-term systems approach to EVD.^4^

## SMART SYSTEMS STRENGTHENING

Much of the problem with Ebola is behavioral, coupled with an underlying deficiency in primary and secondary health care systems. Stopping the outbreak and mitigating future outbreaks depends on improved infrastructure at primary and secondary facilities, as well as rapid behavioral changes including better patient care-seeking behavior, the modification of funeral practices, and the avoidance of bush meat harvesting and consumption. What's needed is flatter, more prevention-focused systems that center on smart primary health care along with more upstream emphasis on things such as health-positive behavior, water, sanitation, and hygiene (WASH), and addressing adverse cultural practices. Implementing organizations and others recognize the need for health systems strengthening but require bi- and multilateral donors to dedicate specific, significant, and sustained resources to help build those systems.^5^^,^^6^

Functional platforms established by long-term investments in family planning, HIV prevention and control, polio eradication, the expanded program for immunization, and malaria control can serve as a foundation for strengthening systems sustainably. Savvy leaders and programmers can capitalize on these opportunities in the near and long terms. Systems strengthening synergies exist within present global health priorities such as laboratory strengthening, human resource development/technical training, logistics/supply chain management, behavior change/IEC, and monitoring/evaluation.^7^

Clearly, systems require an immense amount of specific and sustained inputs and prioritized, strategic approaches. Priorities include a focus on primary preventive and curative care, building on existing platforms, especially HIV/AIDS and family planning, and focusing on positive behavior change. Technical inputs are broad and complex (Box). But systems also require less tangible inputs that concern quality, access, culture, traditional beliefs, education, private-sector engagement, accountability, policy dialogue, and public perceptions concerning fear and distrust of the health care system itself.

**BOX: SYSTEM STRENGTHENING INPUTS**

- Increased numbers of trained, equipped, and compensated personnel
- Supplies, equipment, commodities, and improvements to, and maintenance of, physical plant infrastructure
- Sustainable water, sanitation, electricity, and waste disposal for all facilities
- Appropriate data management and data-based decision-making
- Supply chain management
- Improved access to prevention and curative care for clients
- Effective management at all levels of the system
- Improved behavior change
- Increased engagement of the private sector in service delivery

By definition, systems are interdependent and all elements need strengthening. However, key priorities in lower-income countries include financing, equipping the health workforce, infrastructure support, effective management, and functional partnerships between the public and private sector.

The legacy of vertical programs is that they have built systems platforms to some extent. If we capitalize on those platforms, we can strengthen the systems more broadly. It will take creative programming and piggybacking inputs across the various, more vertical programs that exist today.

Let's not let the momentum and awareness generated by this Ebola outbreak go to waste. Let's strike while the iron is hot and proceed to support better health systems to help mitigate future Ebola outbreaks while limiting the other major health problems rampant in the developing world. We must be proactive rather than reactive.
